# Supplementary material for: IKK/NF-κB Inactivation by Salidroside via Targeting TNF-α for the Treatment of LPS-Induced Colitis
Source: Curr Issues Mol Biol. 2025 Oct 28;47(11):896. doi: 10.3390/cimb47110896 (PMC12651885; doi:10.3390/cimb47110896)
Supplement: Supplementary file 1 [file cimb-47-00896-s001.zip › cimb-3929494-supplementary.pdf]

Supplementary Figure

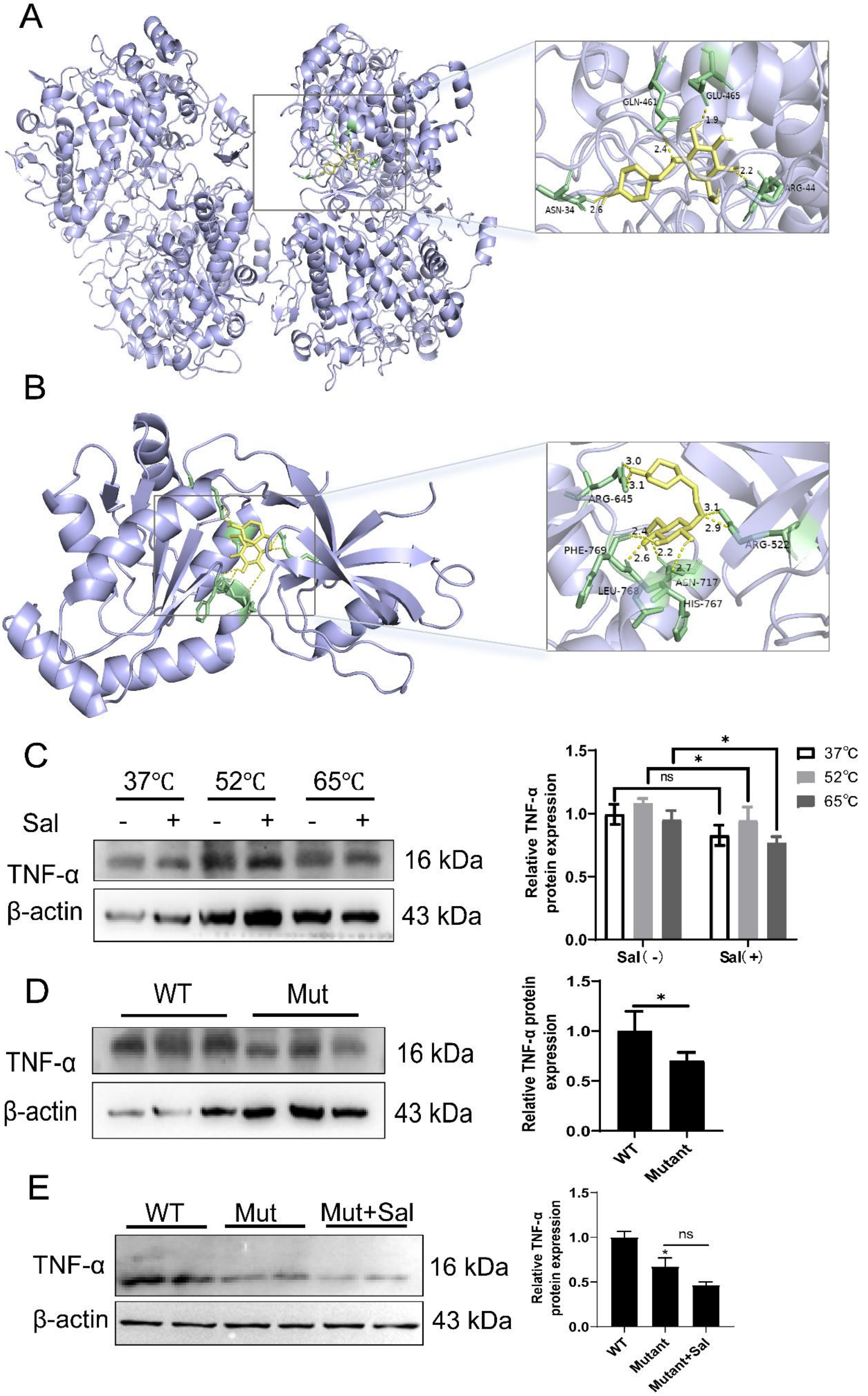

Figure S1. Different amino acids played roles in the binding of Sal with target proteins. A. Molecular docking of salidroside with COX-2. B. Molecular docking of salidroside with ZO-1. C. CETSA of TNF-α protein stability in NCM 460 cells treated with Sal and quantitative analysis of TNF-α protein in NCM460 cell. The values represent the mean ± S.D. ns, no significance, $*p<0.05$, vs control group. D. Western blot analysis of TNF-α protein expression in NCM 460 cells expressing WT or mutant. β-actin served as loading control. $*p<0.05$, vs WT. E. Arg179Ala, Lys188Ala, Tyr191Ala abolished the binding of salidroside with TNF-α. The values represent the mean ± S.D. ns, no significance, $*p<0.05$, vs. WT.

Supplementary Tables

Table S1. RT-qPCR primers for genes associated with inflammatory in cells or mouse colon mice.

Primer name	5'→3'
Human-IL1B-F1	ATGATGGCTTATTACAGTGGCAA
Human-IL1B-R1	GTCGGAGATTCGTAGCTGGA
Human-IL6-F1	ACTCACCTCTTCAGAACGAATTG
Human-IL6-R1	CCATCTTTGGAAGGTTCAGGTTG
Human-β-actin-F1	CTGGCACCCAGCACAATG
Human-β-actin-F1	GCCGATCCACACGGAGTACT
Human-TNF-α-F1	CCTCTCTCTAATCAGCCCTCTG
Human-TNF-α-R1	GAGGACCTGGGAGTAGATGAG
Il-1b（mouse）-F	GCAACTGTTCCTGAACTCAACT
Il-1b（mouse）-R	ATCTTTTGGGGTCCGTCAACT
Il-6（mouse）-F	TAGTCCTTCCTACCCCAATTTCC
Il-6（mouse）-R	TTGGTCCTTAGCCACTCCTTC
Tnf-α（mouse）-F	CCCTCACACTCAGATCATCTTCT
Tnf-α（mouse）-R	GCTACGACGTGGGCTACAG
β-actin（mouse）-F1	GGCTGTATTCCCCTCCATCG
β-actin（mouse）-R1	CCAGTTGGTAACAATGCCATGT

Table S2. Interacting proteins of Sal from SwissTarget Prediction (Top 20).

Target	Common name	Uniprot ID	Target Class	Probability
LXR-alpha	NR1H3	Q13133	Nuclear receptor	0.906315
Androgen Receptor	AR	P10275	Nuclear receptor	0.906315
Cytochrome P450 17A1	CYP17A1	P05093	Cytochrome P450	0.786927
Cytochrome P450 19A1	CYP19A1	P11511	Cytochrome P450	0.740326
Estrogen receptor alpha	ESR1	P03372	Nuclear receptor	0.70743
Estrogen receptor beta	ESR2	Q92731	Nuclear receptor	0.484896
Testis-specific androgen-binding protein	SHBG	P04278	Secreted protein	0.43552
Sterol regulatory element-binding protein 2	SREBF2	Q12772	Unclassified protein	0.320219
Niemann-Pick C1-like protein 1	NPC1L1	Q9UHC9	Other membrane protein	0.320219
Cytochrome P450 51 (by homology)	CYP51A1	Q16850	Cytochrome P450	0.320219
HMG-CoA reductase	HMGCR	P04035	Oxidoreductase	0.287307
Muscarinic acetylcholine receptor M2	CHRM2	P08172	Family A G protein-coupled receptor	0.25429
Acetylcholinesterase	ACHE	P22303	Hydrolase	0.25429
TNF-alpha	TNF	P01375	Secreted protein	0.25429
Serotonin transporter	SLC6A4	P31645	Electrochemical transporter	0.25429
Cytochrome P450 2C19	CYP2C19	P33261	Cytochrome P450	0.25429
Nuclear receptor subfamily 1 group I member 3	NR1I3	Q14994	Nuclear receptor	0.171979
Nuclear receptor ROR-gamma	RORC	P51449	Nuclear receptor	0.130792
Corticosteroid binding globulin	SERPINA6	P08185	Secreted protein	0.114338
Glucose-6-phosphate 1-dehydrogenase	G6PD	P11413	Enzyme	0.114338

Table S3. Statistical analysis of salidroside binding with target proteins.

Drug	PDB ID	target protein	binding energy（kcal/mol）
Salidroside	159278	TNF-α	-6.3
		COX2	-8
		ZO-1	-7.8
